# Urbanization Level and Vulnerability to Heat-Related Mortality in Jiangsu Province, China

**DOI:** 10.1289/EHP204

**Published:** 2016-05-06

**Authors:** Kai Chen, Lian Zhou, Xiaodong Chen, Zongwei Ma, Yang Liu, Lei Huang, Jun Bi, Patrick L. Kinney

**Affiliations:** 1 State Key Laboratory of Pollution Control and Resource Reuse, School of the Environment, Nanjing University, Nanjing, China; 2 Program in Climate and Health, Department of Environmental Health Sciences, Mailman School of Public Health, Columbia University, New York, New York, USA; 3 Jiangsu Provincial Center for Disease Prevention and Control, Nanjing, China; 4 Department of Environmental Health, Rollins School of Public Health, Emory University, Atlanta, Georgia, USA

## Abstract

**Background:**

Although adverse effects of high temperature on mortality have been studied extensively in urban areas, little is known of the heat–mortality associations outside of cities.

**Objective:**

We investigated whether heat–mortality associations differed between urban and nonurban areas and how urbanicity affected the vulnerability to heat-related mortality.

**Methods:**

We first analyzed heat-related mortality risk in each of 102 counties in Jiangsu Province, China, during 2009–2013 using a distributed-lag nonlinear model. The county-specific estimates were then pooled for more urban (percentage of urban population ≥ 57.11%) and less urban (percentage of urban population < 57.11%) counties using a Bayesian hierarchical model. To explain the spatial variation in associations by county, county-level characteristics affecting heat vulnerability were also examined.

**Results:**

We found that the overall mortality risk comparing the 99th vs. 75th percentiles of temperature was 1.43 [95% posterior intervals (PI): 1.36, 1.50] in less urban counties and 1.26 (95% PI: 1.23, 1.30) in more urban counties. The heat effects on cardiorespiratory mortality followed a similar pattern. Higher education level and prevalence of air conditioning were significantly associated with counties having lower risks, whereas percentage of elderly people was significantly associated with increased risks.

**Conclusion:**

Our findings reveal that nonurban areas have significant heat-related mortality risks in Jiangsu, China. These results suggest the need for enhanced adaptation planning in Chinese nonurban areas under a changing climate.

**Citation:**

Chen K, Zhou L, Chen X, Ma Z, Liu Y, Huang L, Bi J, Kinney PL. 2016. Urbanization level and vulnerability to heat-related mortality in Jiangsu Province, China. Environ Health Perspect 124:1863–1869; http://dx.doi.org/10.1289/EHP204

## Introduction

A large number of epidemiological studies have reported adverse impacts of heat exposure on mortality in cities (Anderson and Bell 2009; Basu 2009; Curriero et al. 2002; McMichael et al. 2008), leading to increasing concern regarding heat-related mortality risks in urban areas under climate change (Li et al. 2013; Petkova et al. 2013). Until now it has been generally assumed that urban areas are more vulnerable to heat because of higher exposures caused by the “urban heat island effect” (Hajat and Kosatky 2010). However, heat vulnerability is determined not only by heat exposure, but also by sensitivity and adaptive capacity (Manangan et al. 2014). Nonurban areas with high sensitivity and low adaptive capacity (e.g., limited availability of health resources and air conditioning) could also be vulnerable.

Though very few in number, prior studies that evaluated heat impacts in nonurban areas provide support for the notion that nonurban impacts can be significant (Bennett et al. 2014; Madrigano et al. 2015; Sheridan and Dolney 2003). In Guangdong Province, China, heat-related mortality effects were found to be higher for two rural communities than for two urban communities (Zeng et al. 2014). In contrast, in Bangladesh, heat effects were more pronounced in urban areas than in rural areas (Burkart et al. 2011). This inconsistency warrants further research using sufficient mortality cases covering a large number of urban and rural areas to analyze the effect of urbanicity on heat-related mortality.

Much of the evidence on the heat-related mortality derives from multi-city time-series studies, which provide robust and generalizable results (Anderson and Bell 2009; Basu 2009; Curriero et al. 2002). However, few multi-site studies have been conducted in developing countries such as China (Ma et al. 2014), where the spatial patterning of socioeconomic status, demographic characteristics, and access to health care may be different from developed countries. One international study suggested that people in developing countries were more vulnerable to heat-related mortality risks (McMichael et al. 2008). Although most previous multi-site studies analyzed data in large spatial units, such as metropolitan areas, recent studies have demonstrated spatial variability in heat vulnerability at smaller sub-city scales (Bennett et al. 2014; Reid et al. 2009). Small-scale analyses are crucial for understanding heat vulnerability patterns and designing appropriate local adaptation strategies.

In the present study, we collected mortality data for all 102 counties spanning Jiangsu Province, China, a diverse region that provides a unique opportunity to examine whether heat-related mortality risk differs between urban and nonurban areas and its underlying causes using a multi-site time-series analysis with high spatial resolution.

## Methods

### Data

This study was conducted in 102 counties in Jiangsu Province, China (see Figure S1) with a total population of 73.9 million people in 2010. The studied counties in Jiangsu Province have an average area of 904.9 km^2^. Daily mortality data from 1 January 2009 through 31 December 2013 were collected from Jiangsu Provincial Center for Disease Prevention and Control. Total (non-accidental) and cardiorespiratory deaths for all ages were extracted based on the *International Statistical Classification of Diseases and Related Health Problems, 10th Revision* (ICD-10): total (codes A00–R99) and cardiorespiratory deaths (codes I00–I99, J00–J99). A total of 2 million non-accidental deaths occurred in our study area from 2009 through 2013. To focus on heat-related mortality, we examined the effect of high temperature on mortality in the warm season (May–September).

Daily temperatures at 10 km × 10 km resolution were interpolated from daily weather station observations using the Australian National University Splines (ANUSPLIN) thin plate smoothing software (Hutchinson and Xu 2013), taking into account longitude, latitude, and elevation, as follows:

*Temp_i_ = f* (*lat_i_, lon_i_*) *+ b × Elev_i_ + e_i_*, [1]

where *Temp_i_* is the daily temperature at station *i*; *f*() is the thin plate spline function; *lat_i_* and *lon_i_* are latitude and longitude for station *i*; *Elev_i_* is the elevation of station *i*; *b* is the coefficient for *Elev_i_*; and *e_i_* is the error term at station *i*. The relative humidity was interpolated using a similar method. Daily climate observations for 792 climate stations across China were provided by China Meteorological Data Sharing Service System. Interpolated daily temperatures and relative humidity were first obtained for all of China, and then extracted for Jiangsu Province. We used 10-fold cross-validation to confirm the prediction accuracy of the thin plate smoothing spline method for daily mean temperature [*R* ^2^ = 0.98; root mean squared prediction error (RMSE) = 1.55°C], daily maximum temperature (*R* ^2^ = 0.98; RMSE = 1.83°C), and daily minimum temperature (*R* ^2^ = 0.97; RMSE = 2.13°C) (see Figure S2). We overlaid the county-level mortality data with the gridded temperature and relative humidity data and then computed county-specific daily temperature and relative humidity by computing the mean value within each county area. In sensitivity analyses, we reran key health models using observed daily temperatures in the 21 counties where weather stations were located.

Monthly mean concentration of satellite-based particulate matter with an aerodynamic diameter of ≤ 2.5 μm (PM_2.5_) at 10 km × 10 km resolution was obtained from a previous study (Ma et al. 2016). The monthly mean satellite-based PM_2.5_ was estimated by a two-stage spatial statistical model and had accurate predictions with little bias (*R* ^2^ = 0.73; validation regression slope = 0.91). Using the same method as for daily temperature, monthly mean PM_2.5_ was then aggregated to the county level by deriving the mean value within each county area.

To explain the spatial variation of heat-related mortality risk, county-level characteristics were collected from the 2010 Population Census of China (National Bureau of Statistics of China 2012) and the 2009–2013 Statistics Yearbooks of Jiangsu Province (Jiangsu Statistical Bureau 2009–2013). Variables included population, percentage of urban population, percentage of people ≥ 65 years old, percentage of unemployed people, average years of education, number of air conditioning units per household, number of beds in health institutions per 1,000 people, gross domestic product (GDP), and revenue of local government. To explore the modifying effect of urbanicity on heat-related mortality, we divided all the counties into two groups on the basis of urban fraction: more urban counties (percentage of urban population ≥ median percentage, i.e., 57.11%) and less urban counties (percentage of urban population < 57.11%). For simplicity, henceforth we use “urban” to refer to more urbanized counties, and “nonurban” to refer to less urbanized counties.

### Statistical Analysis

Data were analyzed using a two-stage model. In the first stage, we applied a distributed lag nonlinear model (DLNM) with a quasi-Poisson regression to evaluate the heat–mortality relationship in each county. DLNM can flexibly evaluate the cumulative effects of temperature while accounting for nonlinear effects of temperature at different lag days (Gasparrini 2011). We used a smooth spline function of time to adjust for long-term trends and seasonal variation of mortality. Day of the week was included as a dummy variable. The DLNM for each county used the following formula:

Ln*E*(*Y_t_*) = α + β*Cb.temp_l_* + *ns*(*time*) + δ*DOW*, [2]

where *E(Y_t_)* is the expected daily mortality count on day *t*, *Cb.temp_l_* is a cross-basis matrix for the two dimensions of temperature and lags, *l* refers to the maximum lag days; β and δ are the coefficients for *Cb.temp_l_* and DOW; the natural cubic spline function *ns*() captures the nonlinear relationships between the covariate (time) and mortality; and *DOW* is the dummy variable for day of the week.

We applied natural cubic splines with 4 degrees of freedom (df) for temperature and 4 df for lag days (knots at equally spaced values in the log scale of lags by default). We used a maximum lag of 6 days because previous studies showed that the heat effect usually lasted no more than a week (Anderson and Bell 2009; Wu et al. 2013). As in a previous study (Chen et al. 2015), splines with 3 df per warm season were used to control for long-term and seasonal time trends. Heat effects were calculated as the cumulative relative risk of mortality at 32.27°C (corresponding to the mean value of the 99th percentile for 102 counties) relative to 24.13°C (mean value of the 75th percentile). Sensitivity analyses were used to examine different degrees of freedom for time, temperature, and lag response, alternative maximum lag days, different daily temperature metrics (maximum and minimum temperatures), county-specific temperature thresholds, and controlling for relative humidity.

In the second stage, county-specific estimates across all urban or nonurban counties were combined to estimate the national average risk of urban or nonurban heat-related mortality using a Bayesian hierarchical model (Dominici et al. 2000). As in a previous study (Bobb et al. 2014), a two-level normal independent sampling estimation with uniform priors was used in this analysis. County-level characteristics and satellite-based PM_2.5_ concentrations were then included separately to identify possible effect modifiers of heat-related mortality risk. A more stringent classification of urban types (percentage of urban population < 20.0% for low urbanized, 20.0 to < 40.0% for medium low urbanized, 40.0 to < 60.0% for medium urbanized, 60.0 to < 80.0% for medium high urbanized, and 80.0–100.0% for high urbanized counties) was also tested in sensitivity analyses.

To explore the relationship between urbanicity and heat vulnerability, we computed a heat vulnerability index based on four county-level characteristics that were significant predictors (i.e., effect modifiers) of the county-level heat-related mortality relationship. These included average years of education, percentage of people ≥ 65 years old, number of air conditioning units per household, and number of beds in health institutions per 1,000 people. We first normalized each variable to a range of 0–1 by the Min-Max normalization method. The Min-Max normalization used the following formula:

*X.normalized* = (*X* – *X_min_*)/(X*_max_* – X*_min_*), [3]

where *X.normalized* is the normalized value for variable *X*, *Xmin* is the minimum value for variable *X*, and *Xmax* is the maximum value for variable *X*. Then the normalized variables were multiplied by –1 if they were negatively associated with heat-related mortality risks. Finally, a heat vulnerability index was calculated by summing these normalized variables. Thus, a high score of heat vulnerability index denotes high vulnerability. The associations between heat vulnerability index and heat-related mortality risks were explored using linear regression. To test whether these models were affected by spatial autocorrelation of residuals (Griffith 1987; Kissling and Carl 2008), we used the global Moran’s *I* statistic.

## Results

Table 1 summarizes the daily deaths, mean temperature, and county-level characteristics for all, urban, and nonurban counties in Jiangsu Province, China. Compared with urban counties, nonurban counties had higher total and cardiorespiratory mortality rates. As expected, the daily mean temperature was higher in urban counties than in nonurban areas (16.1°C vs. 15.4°C).

**Table 1:** Summary statistics of temperature, mortality, and county characteristics in Jiangsu Province (2009–2013).

Characteristic	Total (102 counties)	Urban (51 counties)	Nonurban (51 counties)
Daily death counts per county in warm season (May–September)
Cardiorespiratory	5.2 ± 3.4	3.2 ± 2.3	7.3 ± 3.1
Total (non-accidental)	9.9 ± 6.0	6.3 ± 4.3	13.8 ± 5.2
Daily mortality rates in warm season (May–September) (per 100,000 people)	
Cardiorespiratory	0.7	0.5	0.9
Total (non-accidental)	1.4	1.0	1.6
Daily mean temperature (°C)
Mean	15.7 ± 0.9	16.1 ± 0.8	15.4 ± 0.8
75th percentile	24.1 ± 0.7	24.4 ± 0.6	23.8 ± 0.6
99th percentile	32.3 ± 0.8	32.6 ± 0.8	31.9 ± 0.8
County characteristics
Population (10,000 people)	72.5 ± 34.7	63.3 ± 38.3	81.7 ± 28.3
Percentage of people ≥ 65 years old	10.7 ± 2.8	9.2 ± 1.7	12.3 ± 2.8
Percentage of unemployed people	16.1 ± 4.1	14.2 ± 4.3	18.0 ± 2.9
Average years of education	9.6 ± 1.2	10.5 ± 1.0	8.6 ± 0.4
Number of air conditioning units per household	1.4 ± 0.5	1.8 ± 0.6	1.0 ± 0.3
Number of beds in health institutions per 1,000 people	4.0 ± 2.0	5.0 ± 2.2	2.9 ± 1.2
GDP (billion RMB)	38.3 ± 37.0	47.5 ± 42.3	28.9 ± 19.5
Revenue of local government (billion RMB)	2.9 ± 2.7	3.6 ± 3.5	2.2 ± 1.3
We used percentage of urban population ≥ 57.11% as the definition of urban county.			

**Figure 1:**
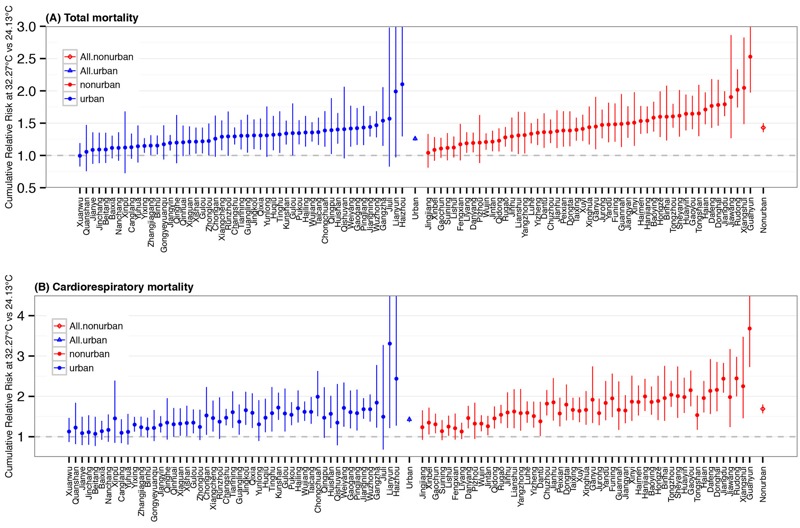
Estimated cumulative relative risks of total (*A*) and cardiorespiratory (*B*) mortality at 32.27°C (mean 99th percentile for 102 counties) relative to 24.13°C (mean 75th percentile) in Jiangsu, China, during 2009–2013. Intervals correspond to the 95% confidence intervals (CIs) for county-specific estimates or the 95% posterior intervals (PIs) for pooled overall estimates. Estimates are shown in order of total mortality risks for urban and nonurban counties.

Figure 1 presents the county-specific cumulative heat-related mortality risks for 102 counties, together with the pooled average risks for all urban and all nonurban counties. Seventy-six counties had significantly increased heat-related total mortality risks and 78 counties had significantly increased heat-related cardiorespiratory mortality risks during the study period. The overall cumulative total mortality risk was 1.43 [95% posterior interval (PI): 1.36, 1.50] in nonurban counties and 1.26 (95% PI: 1.23, 1.30) in urban counties. The overall heat-related cumulative cardiorespiratory mortality risk was also higher in nonurban counties (1.69; 95% PI: 1.58, 1.80), compared with urban counties (1.43; 95% PI: 1.36, 1.50).

Results on effect modification by county-level characteristics are shown in Table 2. An interquartile range (IQR) increase in percentage of people ≥ 65 years old (i.e., 2.9% increase) was associated with a 4.6% (95% PI: 1.6, 7.7) increase in the effect of temperature on total mortality and a 6.2% (95% PI: 1.9, 10.6) increase of the effect on cardiorespiratory mortality. IQR increases in average years of education, number of air conditioning units per household, and number of beds in health institutions per 1,000 people were significantly associated with decreases of 12.0% (95% PI: 7.7, 16.0), 14.1% (95% PI: 9.6, 18.4), and 3.2% (95% PI: 0.4, 6.0) in the effects of temperature on total mortality, respectively, with similar effects for cardiorespiratory mortality risks. No significant effects were found for population, percentage of unemployed people, GDP, and revenue of local government.

**Table 2:** Percent increase (95% PI) in heat-related mortality per interquartile range (IQR) increase in county-level characteristics and air pollutant.

Variables	IQR	Total	Cardiorespiratory
Demographic, social, and economic characteristics
Population	0.5 million	1.6 (–3.1, 6.5)	4.3 (–2.3, 11.3)
Percentage of people ≥ 65 years old	2.9%	4.6 (1.6, 7.7)	6.2 (1.9, 10.6)
Percentage of unemployed people	5.5%	3.1 (–1.1, 7.5)	5.0 (–0.9, 11.2)
Average years of education	2.1 years	–12.0 (–16.0, –7.7)	–15.5 (–20.9, –9.7)
Number of air conditioning units per household	0.9 unit	–14.1 (–18.4, –9.6)	–18.5 (–24.1, –12.6)
Number of beds in health institutions per 1,000 people	1.7 beds	–3.2 (–6.0, –0.4)	–4.5 (–8.2, –0.8)
GDP	24.1 billion RMB	–0.2 (–0.5, 0.1)	–0.2 (–0.6, 0.2)
Revenue of local government	1.7 billion RMB	–1.7 (–3.4, 0.1)	–1.6 (–4.1, 0.9)
Air pollutant: satellite-based PM_2.5_
Average monthly mean level of PM_2.5_	7.8 μg/m^3^	–1.2 (–4.7, 2.5)	–3.2 (–7.8, 1.6)

**Figure 2:**
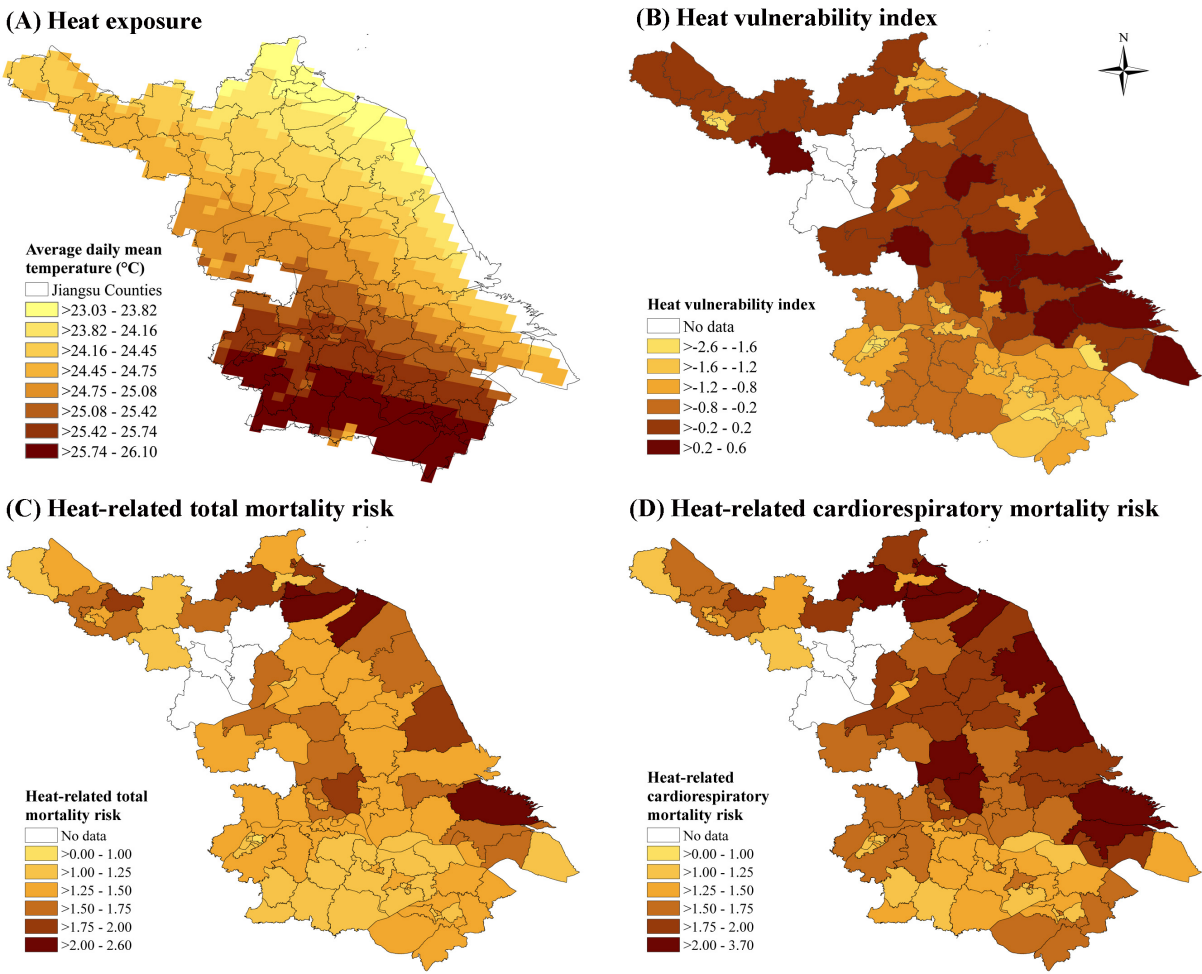
Spatial distribution of heat exposure, vulnerability, and mortality risks in Jiangsu Province, China. (*A*) Average daily mean temperature (°C) over May–September during 2009 to 2013; (*B*) Heat vulnerability index; a higher vulnerability index score indicates a higher vulnerability; (*C*) and (*D*) Heat-related total and cardiorespiratory mortality risks. County-level administrative map was obtained from Provincial Geomatics Center of Jiangsu.

A heat vulnerability index was constructed using the above significant effect modifiers with (–) denoting decreasing vulnerability and (+) denoting increasing vulnerability: (–) average years of education, (+) percentage of people ≥ 65 years old, (–) number of air conditioning units per household, and (–) number of beds in health institutions per 1,000 people. The spatial distribution of county-level heat vulnerability is displayed in Figure 2B. Though heat exposures were generally higher in southern counties than in central and northern counties of Jiangsu Province, heat-related risks for total and cardiorespiratory mortality were both larger in central counties where vulnerability index scores were higher (Figure 2). A significant positive association was observed between the heat vulnerability index and heat-related mortality risk (see Figure S3). Spatial autocorrelation analysis of the regression residuals showed random patterns with *p*-value of Moran’s *I* statistics > 0.05 for both total and cardiorespiratory mortality risks (see Table S2). Thus we did not control for spatial autocorrelation in our models.

**Figure 3:**
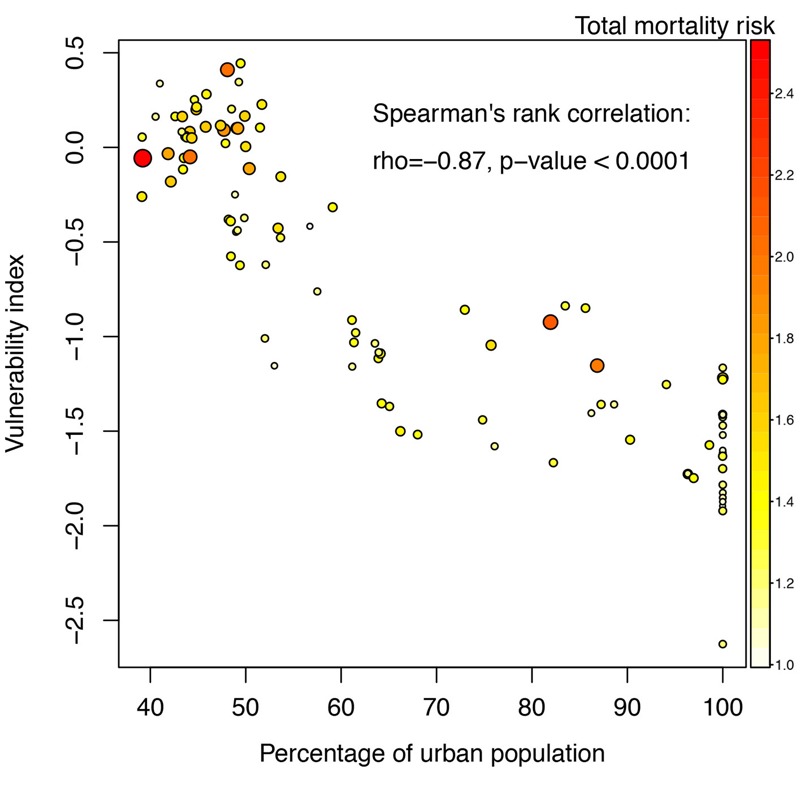
Heat vulnerability index by percentage of urban population for 102 counties in Jiangsu, China. The color and size scale of this scatter plot both represent the heat-related total mortality risk for each county.

Figure 3 shows that the heat vulnerability index was significantly negatively correlated with the urbanicity indicator (percentage of urban population). Counties with higher heat-related mortality risks generally had lower percentages of urban population and higher heat vulnerability index scores (Figure 3; see also Figure S4). Thus, less urbanized counties were generally more vulnerable and had higher heat-related mortality risks in Jiangsu Province.

Associations between heat and daily mortality were not modified by PM_2.5_ concentration (Table 2). Additional sensitivity analyses indicated that our results were robust to modeling choices (see Table S1). Changing degrees of freedom for time, temperature, and lag days generated similar effect estimates (see Table S1). Results remained similar when we used different temperature metrics (i.e., interpolated daily maximum and minimum temperature, directly monitored daily mean temperature), when we controlled relative humidity, and when we used the county-specific temperature percentiles (see Table S1). Using five levels of urbanicity instead of two showed that highly urbanized counties (percentage of urban population ≥ 80.0%) had significantly lower total and cardiorespiratory mortality risks than other urban types with less urbanization.

## Discussion

To the best of our knowledge, this is the first multi-site time-series study to examine the impact of urbanization on vulnerability to heat-related mortality effects in a developing country. Our study provides direct evidence that heat-related mortality risks are not limited to urban areas in China. People living in nonurban counties of Jiangsu Province were more vulnerable and had higher heat-related mortality risks. Our estimated heat effect in urban areas is consistent with a prior multi-site study in China, which found a similar total mortality risk (i.e., 1.21; 95% PI: 1.11, 1.31) comparing the 99th and 75th percentile of temperatures in 17 Chinese cities (Ma et al. 2014). However, the risk we observed in nonurban regions was almost twice as large.

Though numerous studies have addressed the health impacts of high temperatures in urban areas, nonurban areas have been poorly investigated (Todd and Valleron 2015). The effect of urbanization level on heat-related mortality risks remains unclear. A previous study observed more severe heat effects in urban areas of England and Wales (Hajat et al. 2007), whereas another study using high-resolution temperature data found that heat effects were not statistically significantly different between rural and urban areas in the same region (Bennett et al. 2014). Though the definition of urbanicity was somewhat arbitrary in the present study (counties divided into two groups based on the median value of percentage of urban population), findings were consistent with an alternative definition based on five levels of urbanicity (see Table S1). Our findings are consistent with two previous studies in the United States, which found higher percentage increases of heat-related mortality in rural counties than urban counties (Madrigano et al. 2015; Sheridan and Dolney 2003). Similarly, recent studies in France and China provided some evidence that nonurban areas can experience significant heat-related mortality risks (Todd and Valleron 2015; Zeng et al. 2014).

Our findings challenge the general assumption that urban areas are more vulnerable to the impacts of high ambient temperature due to the urban heat island effect and high concentrations of susceptible populations (Kinney et al. 2008). It is generally the case that urban areas have higher ambient temperatures than nonurban areas (Table 1). However, high heat exposure does not necessarily equate to high vulnerability, because heat-related health impacts also depend on other, non-climate vulnerability factors (i.e., sensitivity and adaptive capacity) (Turner et al. 2003). A study in France showed that heat exposure and socioeconomic vulnerability factors had synergistic effects in heat wave–related mortality (Rey et al. 2009).

Recently, heat-related vulnerability indices have been developed and applied to identify and map vulnerable populations and locations (Harlan et al. 2013; Reid et al. 2009). In the present study, we calculated a heat vulnerability index based on non-climate vulnerability factors in order to disentangle the effect of heat exposure and non-climate vulnerability factors on heat-related mortality risks. We found that counties with a higher percentage of urban population had lower vulnerability index scores, suggesting that urban areas had lower proportions of vulnerable populations in Jiangsu Province (Figure 3). Consistently, a mapping study found that vulnerability to heat-related illness extends beyond urban areas in Georgia, USA (Manangan et al. 2014). Because nonurban areas had lower heat exposure, the higher mortality risks in nonurban areas could be attributed to their higher vulnerability.

Although in general our vulnerability index was associated with heat-related mortality risks, there were some counties that did not follow this pattern (see Figure S3). Unique patterns of heat exposure or other unmeasured vulnerability factors may have played a role in deviating these counties from the above vulnerability–risk pattern. This is an area in need of further investigation in the future.

Consistent with previous studies, we found higher heat-related mortality risks in areas with higher percentages of older people (Hajat and Kosatky 2010), people with lower education (Bell et al. 2008), lower prevalence of air conditioning (Anderson and Bell 2009), and insufficiency of hospital infrastructure (Loughnan et al. 2013). Compared with urban counties, nonurban counties in Jiangsu Province had higher levels of all of the above characteristics (Table 1). Older people are vulnerable to heat exposure because of higher physiological sensitivity, preexisting medical conditions, and potential social isolation (Hajat and Kosatky 2010). Education serves as an indicator of socioeconomic status, and people with lower education may not have adequate resources to reduce heat exposure (Chen et al. 2015). Air conditioning is a protective factor against heat-related mortality (Reid et al. 2009); and the insufficiency of hospital infrastructure represents a decreased adaptive capacity (Manangan et al. 2014).

Due to the migration of young people to urban areas, the proportion of older adults has been rising more rapidly in nonurban than in urban areas in China (Gong et al. 2012). From 1982 through 2005, the percentage of the population ≥ 60 years old rose from 7.8% to 13.7% in rural areas and from 7.1% to 12.1% in urban areas, respectively (Cai et al. 2012). By 2030, the aged population in China will reach 21.8% in rural areas and 14.8% in urban areas (Cai et al. 2012), which will pose a serious challenge for China to adapt to the health impacts of climate change. Moreover, because of the migration of rural young people, many older people in rural areas live in “empty nests” (i.e., an elderly person lives alone or with another elderly person). Empty-nest elderly were found to be more socially isolated (Wu et al. 2010), which may also increase their heat-related vulnerability (Reid et al. 2009). In addition, rural elderly people are typically poorer than the urban elderly in China (Cai et al. 2012), which may contribute to their higher vulnerability to heat-related mortality effects.

Although urbanization may amplify future population vulnerability to heat by increasing heat exposures related to the heat island effect (Hondula et al. 2014; Luber and McGeehin 2008; Oudin Åström et al. 2013), population sensitivity and adaptive capacity may also change along with urbanization. A study in Massachusetts found that urbanization was not associated with higher heat-related mortality rates (Hattis et al. 2012). Similarly, our findings suggest that urbanization could lead to lower heat-related vulnerability and decreased mortality risk in the future in Jiangsu, China. In the past three decades, China has experienced rapid urbanization, with the proportion of urban population increasing from 17.9% in 1978 to 52.6% in 2012. A further increase to 60.0% is expected by 2020 (Bai et al. 2014). Urbanization is closely linked to improvements in socioeconomic level in China (Gong et al. 2012). Thus, urbanization-induced heat exposure may be offset by the decreasing socioeconomic vulnerability in the future, leading to a slight increase or even decrease in heat-related mortality risk in China.

Our multi-site analysis enabled us to simultaneously examine the heat-related mortality risks in urban and nonurban areas covering a large spatial area. For the first time, urbanization level was systematically found to be associated with decreased heat-related mortality risks and decreased vulnerability. This work has important implications for heat adaptation planning. The distribution of vulnerability factors by urbanization levels likely differs in China versus more developed counties. Future studies in other locations using a similar design and high spatial resolution are needed to provide a better understanding of the modifying effects of urbanization levels on heat-related mortality risks and more precise information for local adaptations to heat-related health impacts.

Our findings also have implications for studies evaluating the future health impact of high temperatures under climate change. Most such studies use risk estimates for heat-related mortality derived from observed historical data in urban areas (Benmarhnia et al. 2014; Li et al. 2013). By ignoring the differences of risk estimates between urban and nonurban areas along with trends in urbanization, large-scale health impact studies may underestimate or overestimate future heat-related mortality impacts. Furthermore, heat vulnerability patterns may also change over time given the rapid increase of the population of elderly persons. Thus, future vulnerability changes should be considered along with climate change in projecting future heat-related mortality.

Several limitations should be acknowledged in this study. First, there were potential exposure measurement errors because we used interpolated temperatures and satellite-based PM_2.5_ as proxies for actual individual exposure levels. Our interpolated daily mean temperature with a RMSE of 1.55°C may induce exposure measurement error and bias the effect estimates. Averaging interpolated temperature within counties might also result in potential exposure measurement error. However, the exposure measurement error was lessened to some extent because we used fine-scale interpolated temperatures instead of station-based monitored temperatures to better capture the spatial distribution of ambient temperatures within counties. Satellite-based PM_2.5_ estimates also reduced the exposure measurement error to some extent by enabling estimation of PM_2.5_ levels in rural areas where monitoring stations were sparse or nonexistent (Strickland et al. 2016). In addition, this study was conducted in only one province of China. Thus the results may not be applicable to other regions with different climates in China. Third, ozone may modify the association between heat and mortality (Anderson and Bell 2009). However, ozone data were not available during 2009–2012 and thus not included in this study. Fourth, health effects of heat on morbidity were not explored in this study because of the lack of morbidity data. Finally, the nexus between urbanicity and vulnerability to cold temperatures was not addressed here because the present study focused on heat-related health impacts. Future studies using similar approaches should be conducted to investigate the effect of urbanization level on vulnerability to cold-related mortality in order to better project the future temperature-related mortality under climate change.

## Conclusions

We found higher heat-related mortality risks and vulnerability scores in nonurban counties than in urban counties in Jiangsu Province, China. Although most of the research in literature has focused on urban areas, significant mortality risks may also exist in nonurban areas. This evidence is important for policy makers to improve adaptation polices in rural areas of China to prevent heat-related impacts under a changing climate.

## Supplemental Material

(1 MB) PDF
