# Supplementary material for: Urbanization Level and Vulnerability to Heat-Related Mortality in Jiangsu Province, China
Source: Environ Health Perspect. 2016 May 6;124(12):1863–9. doi: 10.1289/EHP204 (PMC5132638; doi:10.1289/EHP204)
Supplement: (1 MB) PDF [file EHP204.s001.acco.pdf]

Environ Health Perspect
DOI: 10.1289/EHP204

Note to readers with disabilities: *EHP* strives to ensure that all journal content is accessible to all readers. However, some figures and Supplemental Material published in *EHP* articles may not conform to 508 standards due to the complexity of the information being presented. If you need assistance accessing journal content, please contact ehp508@niehs.nih.gov. Our staff will work with you to assess and meet your accessibility needs within 3 working days.

Supplemental Material

Urbanization Level and Vulnerability to Heat-Related Mortality in Jiangsu Province, China

Kai Chen, Lian Zhou, Xiaodong Chen, Zongwei Ma, Yang Liu, Lei Huang, Jun Bi, and Patrick L. Kinney

Table of Contents

Table S1. The pooled cumulative relative risk of mortality (95% PI) at 32·27°C (mean 99th percentile for 102 counties) relative to 24·13°C (mean 75th percentile) by varying modelling choices in Jiangsu, 2009-2013.

Table S2. Spatial autocorrelation analysis of residuals of heat-related mortality risks after linear regression on heat vulnerability index using Global Moran's I statistic.

Figure S1. Distribution of 24 weather stations and 102 studied counties in Jiangsu, China.

Figure S2. Density scatter plot of 10-fold cross-validation of interpolated daily temperatures in 792 stations of China, 2009-2013. (A), (B), and (C) are cross-validation results for interpolated daily mean, maximum, and minimum temperatures, respectively. The color scale of this scatter density plot represents the number of data counts in a pixel in the plot. Results of model fitting were also presented. MPE: mean prediction error (°C); RMSE: root mean squared prediction error (°C); RPE: relative prediction error (%). The dashed line is the 1:1 line.

Figure S3. Relationship between heat-related mortality risk and heat vulnerability index in 102 counties of Jiangsu Province, China: (A) total mortality; (B) cardiovascular mortality. County-specific estimates of the overall cumulative total mortality risk at 32·27 °C vs. 24·13 °C were plotted against the county-specific heat vulnerability index scores. The solid lines show the estimated linear associations, and the shaded bands denote 95% Confidence Intervals. Results of Pearson correlation are also presented.

Figure S4. Heat vulnerability index, percentage of urban population, and cardiorespiratory mortality risk for 102 counties in Jiangsu, China. The color and size scale of this scatter plot represents the heat-related cardiorespiratory mortality risk for each county.

Table S1. The pooled cumulative relative risk of mortality (95% PI) at 32·27°C (mean 99th percentile for 102 counties) relative to 24·13°C (mean 75th percentile) by varying modelling choices in Jiangsu, 2009-2013.

	**Urbanity**	**Total**	**Cardiorespiratory**
Main model	Total 102 counties	1.35(1.31,1.39)	1.56(1.49,1.63)
	Urban counties (51)	1.26(1.23,1.30)	1.43(1.36,1.50)
	Nonurban counties (51)	1.43(1.36,1.50)	1.69(1.58,1.80)
Modeling parameters			
Long time and seasonal control: 5 df/warm season	Total 102 counties	1.36(1.31,1.42)	1.55(1.47,1.64)
	Urban counties (51)	1.24(1.19,1.29)	1.37(1.29,1.45)
	Nonurban counties (51)	1.47(1.38,1.56)	1.73(1.60,1.88)
Long time and seasonal control: 4 df for day of the year variable and 2 df for year variable	Total 102 counties	1.28(1.24,1.32)	1.43(1.37,1.50)
	Urban counties (51)	1.18(1.15,1.22)	1.28(1.22,1.34)
	Nonurban counties (51)	1.36(1.30,1.42)	1.51(1.48,1.67)
Df for lag-response:5	Total 102 counties	1.35(1.31,1.39)	1.56(1.49,1.62)
	Urban counties (51)	1.26(1.22,1.30)	1.42(1.35,1.49)
	Nonurban counties (51)	1.43(1.36,1.50)	1.69(1.58,1.80)
Df for lag-response:6	Total 102 counties	1.35(1.31,1.39)	1.56(1.49,1.63)
	Urban counties (51)	1.26(1.22,1.30)	1.42(1.36,1.49)
	Nonurban counties (51)	1.43(1.37,1.51)	1.69(1.59,1.80)
Df for temperature:3	Total 102 counties	1.33(1.30,1.37)	1.54(1.48,1.60)
	Urban counties (51)	1.27(1.23,1.31)	1.43(1.36,1.50)
	Nonurban counties (51)	1.40(1.34,1.46)	1.64(1.55,1.74)
Df for temperature:5	Total 102 counties	1.37(1.32,1.41)	1.58(1.51,1.65)
	Urban counties (51)	1.27(1.23,1.31)	1.42(1.36,1.50)
	Nonurban counties (51)	1.47(1.39,1.55)	1.73(1.62,1.86)
Lag period:3 days	Total 102 counties	1.37(1.33,1.41)	1.55(1.49,1.61)
	Urban counties (51)	1.28(1.24,1.31)	1.43(1.37,1.49)
	Nonurban counties (51)	1.45(1.39,1.52)	1.67(1.57,1.77)
Lag period:10 days	Total 102 counties	1.34(1.30,1.39)	1.56(1.50,1.63)
	Urban counties (51)	1.26(1.22,1.30)	1.43(1.36,1.51)
	Nonurban counties (51)	1.43(1.36,1.50)	1.69(1.59,1.81)
Temperature metrics at 99th percentile vs. 75th percentile			
Interpolated mean temperature using NDVI as an additional covariate	Total 102 counties	1.36(1.32,1.41)	1.58(1.51,1.65)
	Urban counties (51)	1.26(1.22,1.30)	1.42(1.35,1.49)
	Nonurban counties (51)	1.46(1.39,1.54)	1.74(1.63,1.86)
Interpolated maximum temperature	Total 102 counties	1.34(1.30,1.38)	1.55(1.49,1.62)
	Urban counties (51)	1.26(1.22,1.30)	1.43(1.37,1.49)
	Nonurban counties (51)	1.41(1.35,1.48)	1.67(1.56,1.78)
Interpolated minimum temperature	Total 102 counties	1.31(1.27,1.35)	1.51(1.45,1.57)
	Urban counties (51)	1.24(1.20,1.28)	1.39(1.33,1.46)
	Nonurban counties (51)	1.37(1.31,1.43)	1.61(1.52,1.71)

	Urbanity	Total	Cardiorespiratory
Using 21 counties where weather stations located			
Observed mean temperature in 21 counties	Total 21 counties	1.40(1.29,1.51)	1.69(1.51,1.89)
	Urban counties (7)	1.32(1.10,1.58)	1.60(1.30,1.97)
	Nonurban counties (14)	1.44(1.29,1.61)	1.74(1.47,2.06)
Interpolated mean temperature in 21 counties	Total 21 counties	1.41(1.30,1.53	1.71(1.51,1.93)
	Urban counties (7)	1.34(1.15,1.56)	1.65(1.39,1.95)
	Nonurban counties (14)	1.45(1.28,1.65)	1.75(1.44,2.12)
Threshold and reference mean temperature for risk estimates			
Using county-specific 99th percentile temperature vs. 75th percentile temperature	Total 102 counties	1.36(1.32,1.40)	1.57(1.51,1.63)
	Urban counties (51)	1.33(1.28,1.38)	1.52(1.44,1.61)
	Nonurban counties (51)	1.39(1.33,1.44)	1.61(1.53,1.70)
Controlling for satellite-based $PM_{2.5}$			
With monthly $PM_{2.5}$	Total 102 counties	1.37(1.32,1.41)	1.59(1.52,1.67)
	Urban counties (51)	1.27(1.23,1.31)	1.44(1.37,1.52)
	Nonurban counties (51)	1.45(1.38,1.53)	1.72(1.61,1.84)
Controlling for relative humidity			
With relative humidity	Total 102 counties	1.36(1.32,1.40)	1.57(1.50,1.64)
	Urban counties (51)	1.27(1.23,1.31)	1.43(1.37,1.50)
	Nonurban counties (51)	1.44(1.37,1.51)	1.70(1.59,1.81)
Urban types			
Using 5 categories of urban types based on the 20th, 40th, 60th, and 80th percentile of percentage of urban population in 102 counties.	Low urbanized counties (21)	1.50(1.38,1.63)	1.78(1.58,2.00)
	Medium low urbanized counties (20)	1.41(1.30,1.53)	1.63(1.47,1.81)
	Medium urbanized counties (20)	1.32(1.23,1.40)	1.54(1.41,1.69)
	Medium high urbanized counties (20)	1.33(1.26,1.41)	1.54(1.41,1.67)
	High urbanized counties (21)	1.19(1.12,1.25)	1.30(1.19,1.41)

Table S2. Spatial autocorrelation analysis of residuals of heat-related mortality risks after linear regression on heat vulnerability index using Global Moran's I statistic.

Heat-related mortality risk	Global Moran's I	p-value
Total	0.128	0.141
Cardiorespiratory	0.127	0.135

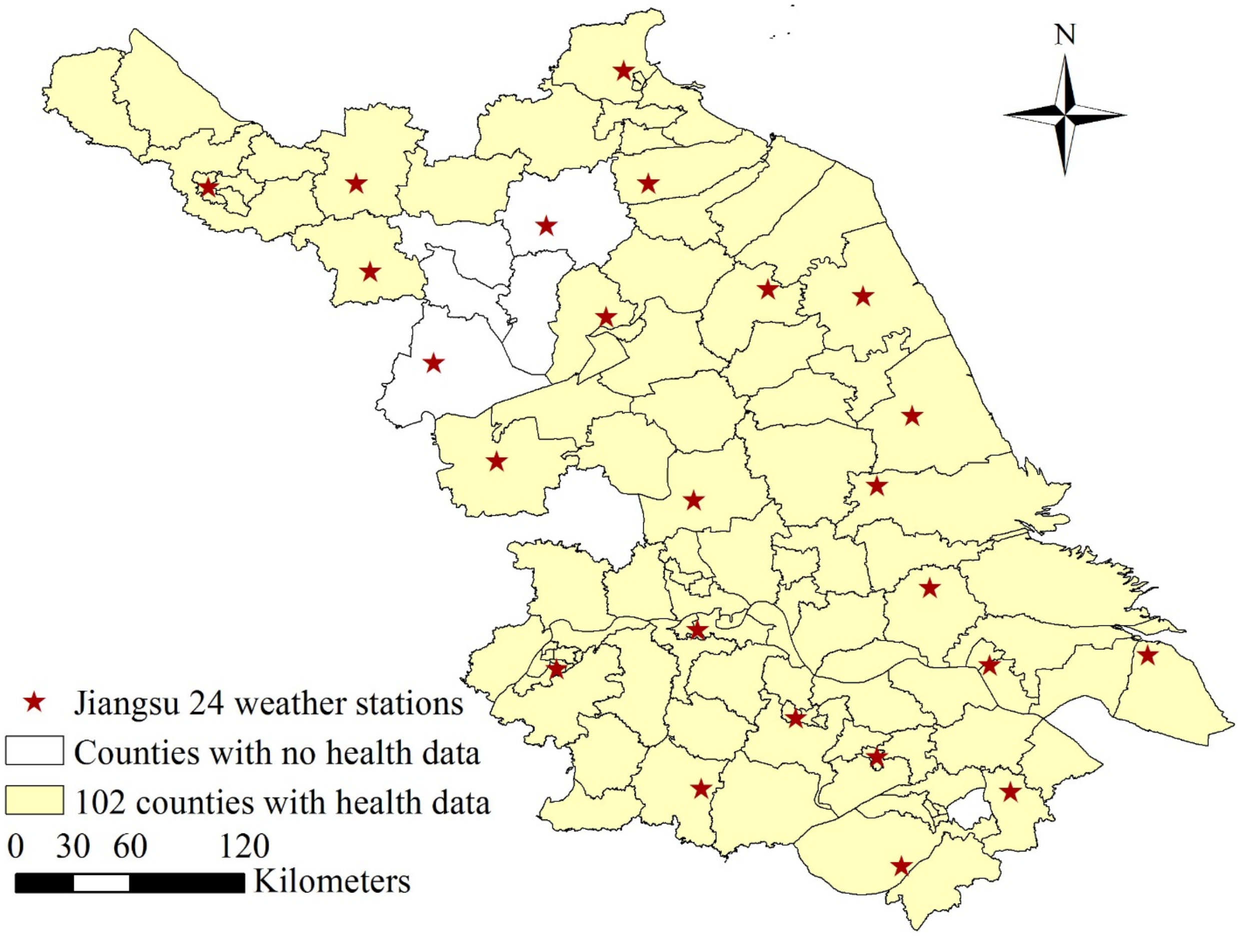

Figure S1. Distribution of 24 weather stations and 102 studied counties in Jiangsu, China.

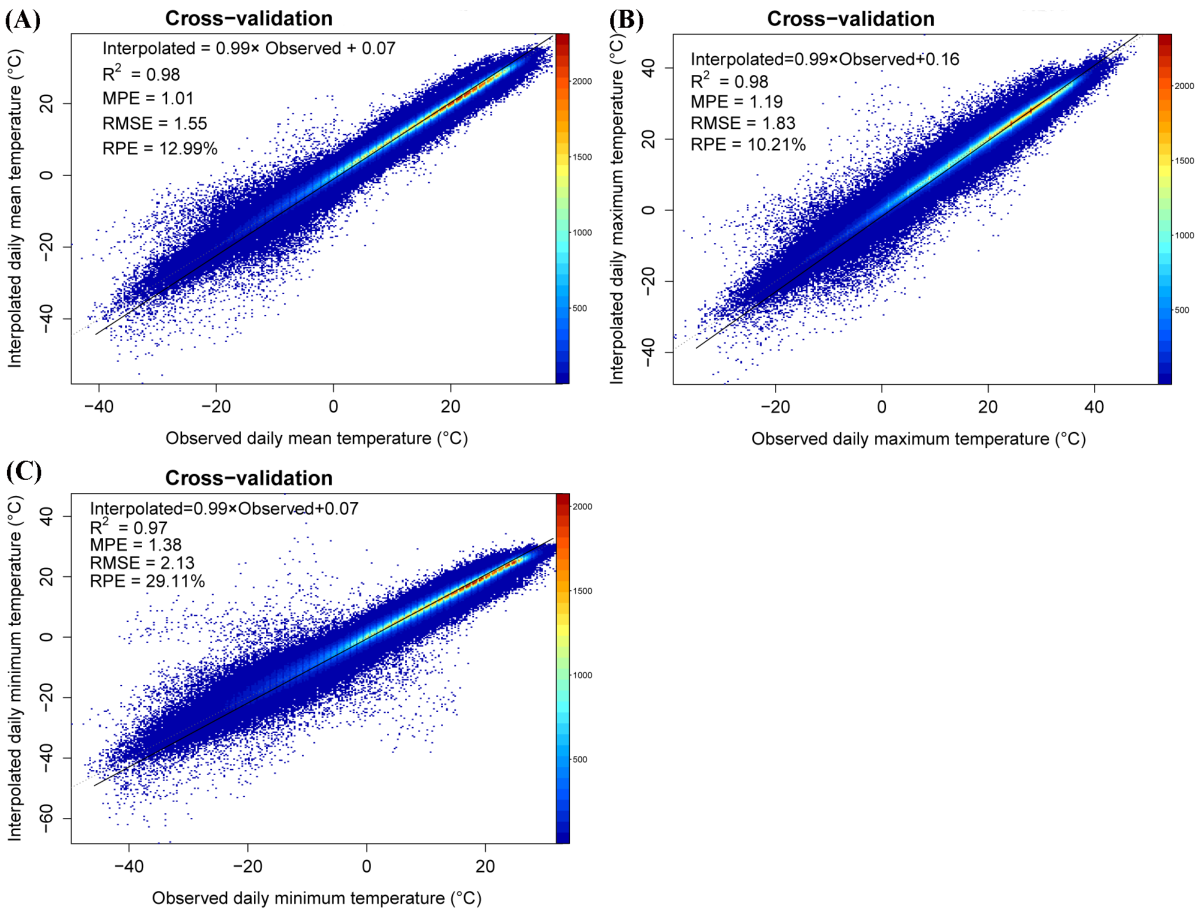

Figure S2. Density scatter plot of 10-fold cross-validation of interpolated daily temperatures in 792 stations of China, 2009-2013. (A), (B), and (C) are cross-validation results for interpolated daily mean, maximum, and minimum temperatures, respectively. The color scale of this scatter density plot represents the number of data counts in a pixel in the plot. Results of model fitting were also presented. MPE: mean prediction error (°C); RMSE: root mean squared prediction error (°C); RPE: relative prediction error (%). The dashed line is the 1:1 line.

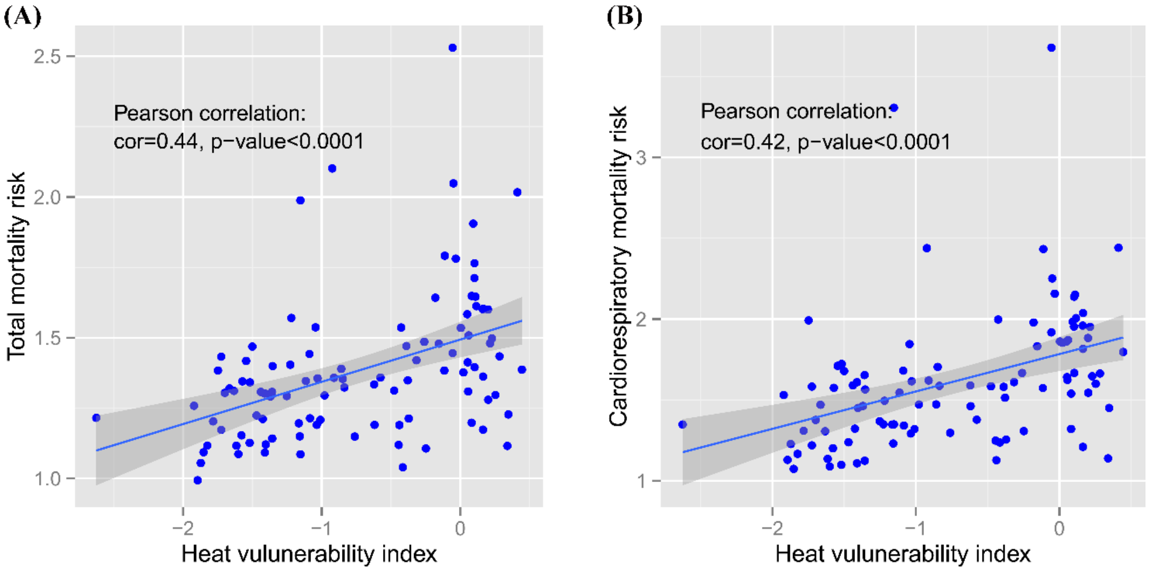

Figure S3. Relationship between heat-related mortality risk and heat vulnerability index in 102 counties of Jiangsu Province, China: (A) total mortality; (B) cardiorespiratory mortality. County-specific estimates of the overall cumulative total mortality risk at 32·27 °C vs. 24·13 °C were plotted against the county-specific heat vulnerability index scores. The solid lines show the estimated linear associations, and the shaded bands denote 95% Confidence Intervals. Results of Pearson correlation are also presented.

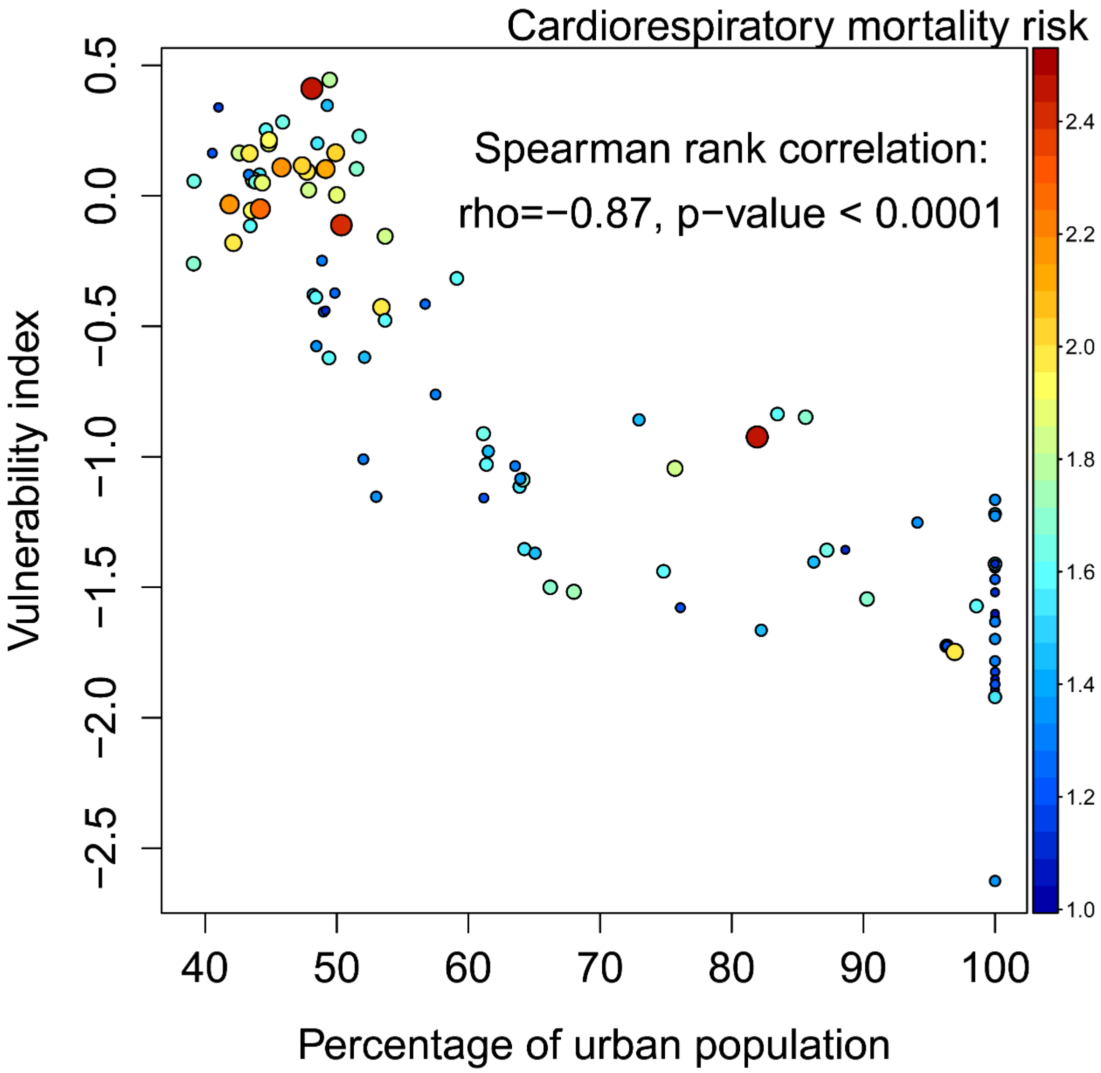

Figure S4. Heat vulnerability index, percentage of urban population, and cardiorespiratory mortality risk for 102 counties in Jiangsu, China. The color and size scale of this scatter plot represents the heat-related cardiorespiratory mortality risk for each county.
